# Epigenetic Effects and Molecular Mechanisms of Tumorigenesis Induced by Cigarette Smoke: An Overview

**DOI:** 10.1155/2011/654931

**Published:** 2011-03-22

**Authors:** Rong-Jane Chen, Louis W. Chang, Pinpin Lin, Ying-Jan Wang

**Affiliations:** ^1^Department of Environmental and Occupational Health, National Cheng Kung University Medical College, 138 Sheng-Li Road, Tainan 70428, Taiwan; ^2^Institute of Molecular Medicine, National Cheng Kung University, Tainan 70428, Taiwan; ^3^Department of Medical Sciences, Kaohsiung Medical University, Kaohsiung 80708, Taiwan; ^4^Division of Environmental Health and Occupational Medicine, National Health Research Institutes, No. 35 Keyan Road, Zhunan Town, Miaoli County 350, Taiwan; ^5^Sustainable Environment Research Centre, National Cheng Kung University, Tainan 70955, Taiwan

## Abstract

Cigarette smoking is one of the major causes of carcinogenesis. Direct genotoxicity induced by cigarette smoke leads to initiation of carcinogenesis. Nongenotoxic (epigenetic) effects of cigarette smoke also act as modulators altering cellular functions. These two effects underlie the mechanisms of tumor promotion and progression. While there is no lack of general reviews on the genotoxic and carcinogenic potentials of cigarette smoke in lung carcinogenesis, updated review on the epigenetic effects and molecular mechanisms of cigarette smoke and carcinogenesis, not limited to lung, is lacking. We are presenting a comprehensive review of recent investigations on cigarette smoke, with special attentions to nicotine, NNK, and PAHs. The current understanding on their molecular mechanisms include (1) receptors, (2) cell cycle regulators, (3) signaling pathways, (4) apoptosis mediators, (5) angiogenic factors, and (6) invasive and metastasis mediators. This review highlighted the complexity biological responses to cigarette smoke components and their involvements in tumorigenesis.

## 1. Introduction

It is known that 90–95% of all cancers are caused by or closely associated with environmental factors and lifestyle. This includes diet (30–35%), cigarette smoking (25–30%), and alcohol consumption (4–6%) [1]. Cigarette smoking is an important risk factor for heart disease, chronic obstructive pulmonary disease, stroke, and acute respiratory diseases. In addition to all these noncancer diseases, it is also highly associated with human cancer development. The International Agency for Research on Cancer (IARC) identified cigarette smoking as the cause of cancer in more organ sites than any other human carcinogens. These include cancers of the lungs, oral cavity, larynx, nasal cavity, esophagus, stomach, pancreas, liver, kidney, urinary bladder, uterine cervix, and bone marrow [2]. There are over 5000 chemical compounds identified in tobacco and 62 of these have been evaluated by IARC as showing “sufficient evidence for carcinogenicity” in either animals or in humans [2, 3]. The major carcinogenic compounds include, but not limited to, radioactive polonium, N-nitrosamines such as 4-(methylnitrosaminao)-1-(3-pyridyl)-1-butanone (NNK), polycyclic aromatic hydrocarbons (PAHs) (e.g., benzo[a]pyrene (BaP)), and benzene [4]. A fine review on this aspect has been presented by Hecht in 2006 [5]. 

The carcinogenesis process is complex. Multistep processes of genetic and molecular defects have taken place before the manifestation of cancer [6]. Traditionally, there are three basic stages of carcinogenesis: initiation, promotion, and progression [7]. Carcinogenesis process is usually accompanied by changes in structure and function of central genomic information coded in the DNA leading to various oncogene activations and tumor suppressor gene inactivations [8]. In addition, multiple signaling pathways may also be deregulated during the process of cancer development. Cancer growth also requires molecular changes that either affect the tumor cells themselves or alter the interaction between tumor cells and their surrounding stromal environment or the immune system. These events may eventually lead to tumor growth, invasion, and metastasis. 

Cigarette smoke components have been reported to promote tumorigenesis by several mechanisms involving all three stages of carcinogenesis [5]. Genotoxic agents in cigarette smoke induce DNA damage through several mechanisms including gene point mutation, deletions, insertions, recombinations, rearrangements, and chromosomal aberrations. PAHs and nitrosamines are two of the most abundant genotoxic components in cigarette smoke. In addition to genotoxic effects, nongenotoxic effects of cigarette smoke are also extremely important. These effects can also act as modulators which alter cellular functions including cell proliferation and cell death. While synergistic effects of genotoxic carcinogens are known to occur, interaction between non-genotoxic (epigenetic) factors and genotoxic agents may also synergistically increase the risk for carcinogenesis [9]. The genotoxicity leading to carcinogenesis has been extensively reviewed in recent years [9–11]. In this present review, aside from a brief overview on the genotoxic effects of cigarette smoke components, we will provide a more extensive review on the non-genotoxic mechanisms of carcinogenesis by cigarette smoke or its components. 

## 2. The Three Carcinogensis Steps Affected by Cigarette Smoke

**Step 1 (Initiation of Carcinogenesis)**

Carcinogenesis may be the result of chemical or biological insults to normal cells through multistep processes that involves genomic changes (initiation of cancer development). Such changes eventually may also lead to cancer promotion and progression [12]. Some of the cigarette smoke components can act directly on DNA, but many require enzyme conversion before becoming carcinogenic [10, 11]. Most of such “conversions” involve metabolic changes via cytochrome *p*450s (*P*450s) such as *P*450s 1A2, 2A13, 2E1, and 3A4 to form the electrophilic entities that can bind to DNA to form DNA adducts. Such adduct formation is usually at the adenine or guanine sites of the DNA and lead to mutations such as those observed in the *KRAS* oncogene in lung cancer or those in the *TP53* gene in a variety of cigarette smoke-induced cancers [13, 14]. These mutation represent the so-called initiation step of carcinogenesis [15].

4-(Methylnitrosamino)-1-(3-pyridyl)-1-butanone (NNK) and *N*′-nitrosonornicotine (NNN) are the most potent tobacco-specific nitrosamines in tobacco products and cigarette smoke. These compounds are formed from tobacco alkaloids like nicotine during the curing process of tobacco and are important tobacco carcinogens that can affect different tissues depending on the specific nitrosamines or their metabolites involved [5, 10]. NNK is a potent lung carcinogen but can also induce liver and nasal cancers. NNN has been shown to be carcinogenic to esophagus, nasal cavity, and respiratory tract in laboratory animals [16]. In humans, metabolites derived from NNK and the metabolites of NNK can also be identified in the smoker's urine [17].

Benzo[a]pyrene (BaP), one of the PAHs, is classified as a Group 1 carcinogen to humans [3]. It has been shown to have strong association and tumor-induction potentials in lungs, trachea, and mammary glands [5]. The carcinogenic potency of BaP has been demonstrated to be related to its metabolites which form DNA adducts with site-specific hotspot mutation in the *p53* tumor suppressor gene. Positive correlations of such adduct formation and tumor are indeed found in the lung cancer tissues of cigarette smokers [18].

These findings indicate that DNA mutations are increased in both tumor and nontumor bearing tissues of smokers. However, it must be pointed out that DNA adduct formations induced by cigarette smoke still cannot fully represent all the risk factors for cancer development in cigarette smokers [19]. For example, while there is higher incidence of pancreatic cancer in cigarette smokers than nonsmokers [20]. Assays for NNK metabolites in pancreatic cancer tissues in humans showed no significant difference between smokers and nonsmokers [21]. Thus, it is apparent that NNK-induced DNA adducts alone are not solely responsible for the pancreatic cancers in cigarette smokers. Nevertheless, NNK and its metabolite, NNAL (4-(methylnitrosamino)-1-(3-pyridyl)-1-butanol), are the only environmental carcinogens known to induce pancreatic cancer in animal models [22]. Thus, the contribution of NNK to pancreatic cancer in cigarette smokers still cannot be ignored. Furthermore, it is suggested that, in addition to DNA damage, synergistic interactions between DNA reactivity and epigenetic actions such as increased cell proliferation induced by NNK or by other chemicals in cigarette smoke may be needed for actual cancer development in such patients [23, 24]. There is indication that cigarette smoke carcinogens or cocarcinogen, such as nicotine, may also play a direct role to enhance cancer promotion and progression in human cancers after cancer development [25]. Such genotoxic mechanisms for cancer initiation and carcinogenesis by cigarette smoke components are well covered and discussed in several excellent reviews [5, 10, 11, 26–28]. Readers are encouraged referring to them. For the remaining portion of this article, we will provide more information on the non-genotoxic (epigenetic) mechanisms involved in cancer promotion and progression via cigarette smoke.

**Step 2 (Cancer Promotion)**

Cancer promotion is characterized by deregulation of signaling pathways which control cell proliferation, apoptosis, and so forth, [29]. It is believed that although there are various genetic pathways which may lead to cancer development or cancer behaviors, there are certain hallmark capabilities or mechanisms which are commonly shared by all tumors. In the following discussion, we will describe each mechanism with illustrated examples.

### 2.1. Effects of Cigarette Smoke on Self-Sufficiency in Growth Signals

Normal cells need mitogenic growth signals to induce proliferation. These signals are transmitted into cells by receptors that bind distinct signaling molecules. In cancer cells, the receptors which transduce growth signals into cells are targets of deregulation during tumorigenesis. Receptor overexpression allows cancer cells to become hyper-responsive to low levels of growth factors that generally are not sufficient to trigger proliferation in normal cells [29]. Nicotine, a major component of cigarette smoke, is known to be a chemical that plays an important role in carcinogenesis in cigarette smokers [30]. Nicotine behaves like those growth factors which exert their biological functions mainly through the nicotinic acetylcholine receptors (nAChR) [31], *β*-adrenoceptors (*β*-AR) [32] or epidermal growth factor receptor (EGFR) [33]. The functions of these receptors are cell-type specific and the expression level and receptor sensitivity can be modified by nicotine. Obviously, alterations in either the receptor expressions or sensitivity play an important role in cigarette smoke-induced carcinogenesis [34–36]. 

Recent study by Lee et al. reported that *α*9 nAChR expression in human breast tumors is elevated in advanced stages of breast cancer and plays important roles in human breast carcinogenesis [37]. Nicotine has been shown to mediate *α*9 nAChR signaling and upregulate cyclin D3 expression in breast caner cells and breast cancer tissues [38]. Furthermore, it is also found that activation of the expression of *α*9 nAChR by nicotine is through AKT signaling [39] and activation of *α*9 nAChR signaling would elevate the phosphorylation status of adhesion molecule which plays a role in cancer metastasis [40]. Proliferation of mesothelioma cells is also found to be enhanced by nicotine [41]. This enhancement has been shown to be via *α*7 nAChR with activation of ERK1/2 cascade as well as induction of NF-*κ*B and Bad phosphorylation. All these events eventually lead to inhibition of apoptosis [41] and increase of cancer risk. These findings were further supported by Wada et al. [42] who observed that nicotine promoted cell proliferation via *α*7 nAChR mediated p44/p42-MAPK activation. Moreover, in our own study, we also reported that nicotine induced human bladder cells proliferation through ERK1/2 and Stat3 signaling downstream of *α*7 nAChR and *β*-adrenoceptors (*β*-AR) [43]. In sum, all these studies indicate that nicotine, an important ingredient of cigarette smoke, promotes cellular proliferation which plays a critical role in carcinogenesis.

Other than nicotine, nitrosamines, such as NNK and NNN, also induced cancer cells growth through nAChR. NNK induced carcinogenesis by binding to nAChR especially for *α*7 nAChR, whereas the biological impact of NNN is mainly modulated by *α*4/*β*2 nAChR [8, 44–46]. It has been demonstrated that nicotine or NNK stimulated lung cancer cell proliferation via *α*7 nAChR with activations of PKC, RAF1, AKT, ERK1/2, and transcription factors such as JUN, FOS, and MYC [47–49]. Question has been raised concerning the possibility that specific nAChR subunit upregulated by nicotine or NNK may be tissue specific or dependent. For instance, with nicotine or NNK, *α*7 nAChR is the primary nAChR subunit which mediates tumorigenesis in lungs giving rise to pulmonary squamous cell carcinoma and mesothelioma [36]. On the other hand, *α*9 nAChR is more associated with breast cancer [37]. Thus, the specific types of nAChR expressed in cancer cells may be considered as useful molecular targets for potential clinical therapy [50]. However, most of the nAChR present in cancer cells are still not functionally characterized yet. Future study will be needed to understand the functions of different nAChR subtype in cancer cells and the downstream signal pathways involved in tumorigenesis. 

In addition to nAChR, a number of studies indicated that nicotine and NNK might also exert their biological activities through activation of receptors such as *β*-adrenoceptors (*β*-AR), EGFR, or insulin-like growth factor receptor (IGFR) or transactivation by nAChR signaling. It has been demonstrated that *β*-AR activation promotes the growths of various adenocarcinoma. For example, NNK can stimulate HT-29 cell proliferation through *β*-AR followed by cyclin AMP elevation and COX-2 expression [51]. Consistently, NNK stimulates the growth of pulmonary adenocarcinoma *in vitro* and *in vivo* via the release of arachidonic acid through COX-2 and 5-lipoxygenase (5-LOX) pathways that are mainly regulated by *β*-AR [52]. In another study by Schuller and Cekanova, NNK is reported to stimulate *β*2-AR receptor pathway (including PKA, cAMP, CREB) and transactivate EGFR pathway (such as Raf-1/ERK1/2 signaling) in the development of lung cancer [53]. It has also been reported that antagonists of *β*-AR can inhibit the development of NNK-induced lung adenocarcinoma [52]. Such antagonists are also found to be effective in reducing the stimulatory effects of nicotine on PKC, ERK1/2 activations, COX-2 expression, and gastric cancer cell proliferation [54]. Elevation of noradrenaline by nicotine via *α*7 nAChR up-regulation leading to significantly enhanced growth and angiogenesis in both gastric cancer and colon cancer has also been demonstrated [55]. Various investigators have also shown increases in neurotransmitters lead to *β*-AR activation, transactivation of EGFR, and the release of EGF [32, 54, 56]. Thus, an interrelationship between nAChR and neurotransmitter is apparent. Indeed, our recent investigation provided compelling evidence that chronic nicotine exposure induced release of noradrenaline via *α*4/*β*2 nAChR activation followed by *β*-AR transactivation. Our study further demonstrated that blocking of *β*-AR with antagonist reversed the nicotine-induced cellular proliferative and chemoresistance [57]. 

Al-Wadei et al. first reported that nicotine contributes to the development of smoking-related pancreatic ductal adenocarcinoma (PDAC) with elevated levels of stress neurotransmitters (adrenaline and noradrenaline) and induction of cAMP, pCREB, and pERK1/2, and inhibition of *γ*-aminobutyric acid (GABA) [58]. GABA has been reported to possess tumor suppressor function suppressing both *β*-AR stimulated PDAC growth and migration* in vitro* [59]. However, while GABA is suppressed in PDACs, noradrenaline, PKA, p-CREB, and pERK1/2 in these tissues are overexpressed. A reduction of GABA by NNK is observed in lung adenocarcinoma [60]. These authors suggested that nicotine and NNK may contribute to the development of PDAC in smokers by suppression of GABA with induction of stress neurotransmitters [61]. Schuller et al. further proposed that nicotine induces the release of stress neurotransmitters through activation of *α*7 nAChR and inhibits release of GABA via inhibition of *α*4/*β*2 nAChR [61]. It is now believed that the stress neurotransmitter released via nAChR activation plays an important role in smoking-associated tumorigenesis [62]. However, the precise mechanisms involved in the regulation and the function of neurotransmitter released by nicotine and NNK are still uncertain. Future research on this area is encouraged. 

It has also been shown that NNK can promote *β*-AR-mediated transactivation of EGFR followed by ERK1/2 phosphorylation leading to an increased proliferation in pancreatic cancer cells [63]. NNK is also reported to induce endogenous IGFR which is associated with the development of lung tumors [64]. Huang et al. also indicated that both activation of thromboxane A2 (TxA2) receptor and synthesis of TxA2 play critical roles in NNK-promoted lung cancer cell proliferation. TxA2 activates the transcriptional factor CREB through both ERK and PI3K/AKT pathways, which may also lead to PCNA and Bcl-2 overexpressions and cell proliferation [65]. These studies provide valuable information on the mechanisms which involve in proliferative signaling stimulated by nicotine and NNK through activation of nAChR, *β*-AR and other growth factor receptors in cancer cells. Triggering such receptors by cigarette smoke would further lead to rapid cell proliferation, cellular migration, invasion, and metastasis. In short, these investigations on the nAChR, and nAChR transactivated with other receptors represent the pivotal role in regulating multiple cellular cascades in general cell functions and in carcinogenesis.

Nicotine is also known to influence signal transducers and activators of transcription 3 (Stat 3) which is an important signal transducer mediating signaling by numerous cytokines, growth factors, and oncoproteins [66]. Findings from our own laboratories indicate that nicotine induces bladder cancer cells proliferation through *α*7 nAChR, *α*4*β*2 nAChR, and *β*-AR followed by activation of ERK1/2 and Stat 3 [43]. Stat3 signaling further enhanced NF-*κ*B activation, cyclin D1 overexpression, and cell cycle progression [43]. Moreover, we also revealed that prolonged stimulation by nicotine upregulated *α*4/*β*2 nAChR and *β*-AR followed with activation of Stat 3 leading to significant increase in chemoresistance in cells from bladder cancers [57]. 

In recent years, nongenotoxic actions of PAHs have gained increasing attentions. The biological effects of PAHs are mainly mediated via aryl hydrocarbone receptor (AhR). Through AhR, PAHs can then trigger ERK1/2 activation and signaling in hepatic “stem cell-like” epithelial cells [67, 68]. Other PAHs, such as benz(a)anthracene (BaA), has also been found to increase DNA synthesis and promote G1-S progression in serum deprived MCF-7 cells [69]. BaP has been shown to increase incidence of tumors in estrogen-responsive rodents, suggesting that it may also affect ER-mediated signaling [70]. PAHs can have actions which mimic those of estrogen. Some investigators believed that the estrogenic property of PAHs may be responsible for the induction of cell proliferation. BaP and BaA have been reported to act as estrogens that stimulate and initiate the ER-mediated transcription and cell cycle progression and enhance ER*α* phosphorylation [70]. On the other hand, there is also indication that the estradiol-dependent cell growth of MCF-7 cells can be inhibited by BaP and BaA [71, 72]. Thus, the actions of PAHs on estrogen-dependent cell proliferation are still controversial. Further studies are needed to elucidate more on the roles of PAHs in carcinogenicity. 

### 2.2. Effects of Cigarette Smoke on Antigrowth Signals

In normal tissues, the antigrowth signals operate to maintain cellular quiescence and tissue homeostasis. Antigrowth signals can block proliferation by forcing the cell cycle progression into the quiescent (G0) state. The cell cycle transition from G1 to S phase is the key regulatory step in the cell cycle and is mainly regulated by CDK4/6-cyclin D and CDK2-cyclin E complexes. These complexes induce Rb phosphorylation and liberate E2Fs allowing cell proliferation to occur [73]. Disruption of the Rb pathway would therefore render cells insensitive to antigrowth factors [29]. Nicotine has been reported to induce binding of Raf-1 to Rb with activation of cyclins and CDKs as well as inactivation of Rb [74]. Via activations of nAChR and *β*-AR, nicotine and NNK both exhibit mitogenic properties by inducing cyclin D1 overexpression leading to G1/S transition and increasing cell cycle progression [49, 75, 76]. NNK can also stimulate normal human lung epithelial cells proliferation through NF-*κ*B and cyclin D1 upregulation in an ERK1/2-dependent pathway [75]. In our own laboratory, we have also demonstrated that nicotine-induced cyclin D1 overexpression is regulated via Stat3, ERK1/2, and NF-*κ*B-dependent pathways in bladder cancer cells [43]. 

Other study also shows that PI3K/AKT-dependent cellular proliferation is also enhanced in response to NNK [49]. The PI3K/AKT pathway is critical in cancer cells because it influences tumorigenesis, tumor growth, and therapeutic resistance [77]. The PI3K/AKT activation is documented in both NNK-treated A/J mice and in human lung cancers from smokers [48]. It also plays a role in NNK-induced cell transformation, proliferation, and metastasis [48]. It has been suggested that AKT and NF-*κ*B may serve as key targets for nicotine or NNK stimulation in the development of lung cancer [49]. West et al. also reported that BEAS2B cells treated with NNK for eight-week period increased cellular proliferation through activation of PI3K/AKT pathways [48]. However, PI3K/AKT activation does not always occur in all cancer cells induced by nicotine. Our previous study indicates that nicotine induced bladder cancer cell proliferation through Stat3 and ERK1/2 signalings instead of via AKT pathway [43]. All these investigations suggest that nicotine or NNK can activate ERK1/2, Stat3, or AKT signaling to interrupt the antigrowth signals leading to enhanced cell cycle progression and cancer promotion. It is important to remember that cigarette smoke components other than nicotine or NNK may also impede on antigrowth mechanisms enhancing cancer development and promotion. Such area of research also deserves focus in the future. 

### 2.3. Antiapoptotic Effects of Cigarette Smoke

Apoptosis plays an important role in controlling normal development, homeostasis, and immune defense via elimination of redundant or abnormal cells in the organism [78]. Failure in cell elimination (reduction of apoptosis) may lead to undesirable cell survival and unchecked cell growths. Resistance to apoptosis is often seen in cancers where cancer cells tend to lose their proapoptotic potentials because of gene mutations. The most important gene mutations include tumor suppressor genes such as *p53*. Nicotine has been shown to inhibit apoptosis induced by tumor necrosis factor (TNF), by ultraviolet (UV), radiation, or by chemotherapeutic drugs such as cisplatin, vinblastine, paclitaxel, and doxorubicin [79]. This antiapoptotic action has been shown to be via PI3K/AKT, Raf/MEKK/ERK1/2, NF-*κ*B, Bcl-2, Bax, Bad, or surviving [23, 80–82]. West et al. demonstrated inhibition of apoptosis and promotion of proliferation in human bronchial epithelium cells by NNK are induced via activation of *α*3/*α*4 nAChR followed by upregulation of AKT, mitogen-activated protein kinase (MAPK), and PKC pathways [48]. Similar results are also observed by Xu and coworkers showing that both AKT and survivin pathways are involved in anticisplatin-induced apoptosis by nicotine [79]. Indeed, drug-induced enhancements of p53 and p21 expressions are shown to be suppressed by nicotine. This anti-apoptotic mechanism is mediated through *α*3 nAChR [83]. Our recent study also indicated that long-term nicotine treatment activated *α*4/*β*2 nAChR and *β*-AR leading to reduction of apoptosis induced by cisplatin or paclitaxol [57]. Consistently, Zhao et al. also reported that nicotine induced up-regulation of Mcl-1 phosphorylation though ERK1/2 via *β*-AR activation with increased chemoresistance (anti-apoptosis) of human lung cancer cells [84]. Other investigators also indicate that NNK can prevent cell apoptosis by modulating the anti-apoptotic Bcl-2 and c-Myc proteins [23]. Heme oxygenase-1 (HO-1) is a protein induced during oxidative stress. It is found to be associated with cellular proliferation and is elevated during the developments of certain malignant tumors such as gastric and thyroid cancers [11–13]. Comparing the HO-1 in lung tissues of smokers and nonsmokers, Li et al. noticed that the expression of HO-1 is significantly increased in both tumor and nontumor tissues of smokers. These studies further revealed that NNK or its metabolites probably induce oxidative stress in lung tissues with elevation on stimulates the expression of HO-1. Such event is through ERK and NF-*κ*B activation and Bad phosphorylation induction leading to eventual apoptosis inhibition [11, 85]. 

Cell proliferation and apoptosis can also be modulated by the peroxisome proliferator-activated receptors (PPARs). PPARs are members of nuclear hormone receptor superfamily of ligand-dependent transcription factors. The major PPAR isoforms are *α*, *β*/*δ*, and *γ* [86]. PPAR*β*/*δ* is expressed in most tissues and has been reported to be associated with cancer growths, especially those in liver, colon, breast and lungs [87–89]. Sun et al. reported a novel mechanism that nicotine increases PPAR*β*/*δ* expression through *α*7 nAChR follow by PI3K/mTOR activation leading to enhanced lung tumor cells proliferation [90]. In contrast to PPAR*β*/*δ*, activation of PPAR*γ* by its ligands induces apoptosis and inhibits cell proliferation [91]. Thus, an intact PPAR*γ* levels or its activation is needed to reduce cancer risk (anti-apoptosis and cell proliferation). Interestingly enough, activation of PPAR*γ* is found to be defective in lung cancers [92]. Furthermore, a significant reduction in the transcriptional activity of PPAR*γ* and its endogenous ligands, including 15-S-Hydroxyeicosatertraenoic acid (15(S)-HETE) and 3-S-hydroxyocatadecadienoic acid (13(S)-HODE), are found reduced in lung tissues of NNK-treated mice. Indeed, lung tumors developed in these mice later. Yuan et al. further suggested that the reduction of 15(S)-HETE and 13(S)-HODE may enable lung cells to be more resistant to apoptosis by NNK and facilitate tumor development in the animals [93]. 

In contrast to nicotine or NNK, PAHs induce either apoptosis or antiapoptosis in mammalian cells [94, 95]. For instance, BaP is known to induce signaling through IGFR and increases cell survival through PI3K activation in human mammary epithelial cells [68]. Solhaug et al. reported that both AKT and ERK1/2 act as anti-apoptosis signals leading to Bad phosphorylation. However, BaP can also induce apoptosis through p53 and p21 signaling in the same model [96]. The results suggest that BaP is capable in stimulating both apoptosis and anti-apoptosis signals. Teranishi et al. reported that light-irradiated BaP (LBaP) inhibited apoptosis through production of ROS from degraded BaP [97]. This anti-apoptotic signal induced by BaP in combination with DNA damage would increase the possibility of cell survival and producing mutations. Thus, while the apoptotic signal of BaP induces cell death (cytotoxicity), the anti-apoptotic signals of BaP play an important role in cell proliferation and carcinogenesis. The precise factors influencing either apoptotic or anti-apoptotic outcome are still unclear. The anti-apoptosis mechanisms induced by components of cigarette smoke are obviously quite complex. It is evident that evading apoptosis plays a critical role in cigarette smoke-induced tumorigenesis and chemoresistance. Further explorations are very much needed. New understandings on the molecular target regulating the apoptotic and anti-apoptosis machineries by cigarette smoke could provide novel strategies for drug development with substantial therapeutic benefits.

### 2.4. Effects of Cigarette Smoke on Replicative Lifespan

When a cell population has progressed through a certain number of doublings (replications), they would normally stop growing and enter into a process called “senescence”. Tumor cells, however, appeared to have limitless replicative potentials (immortalization) during tumor progression [29]. Telomeres, which define the end segments of chromosomes, consist of short, tandemly repeated DNA sequences (TTAGGG)*n* together with associated proteins. They represent important devices in controlling cell divisions and proliferations. Small amount of these end DNA sequences may be lost during each cell cycle as a result of incomplete DNA replication. However, de novo additions of TTAGGG repeats by the enzyme telomerase may compensate for this loss [98]. Thus, telomerase plays an important role in the maintenance of the telomere ends in normal cells. Ectopic expression of telomerase would immortalize the cells. 

By using human tissue samples, Yim et al. reported that there are different distributions of the telomerase activity between smokers or ever-smokers and non-smoker. A strong correlation between telomerase activity and the number of packs years smoked can be established among these subjects indicating that there is an association between tobacco exposure and telomerase activity in the human bronchial epithelium. Increased telomerase activity would extend the “lifespan” of cells and put these cells to be at higher risks for malignant transformation and carcinogenesis [99]. Similar finding is reported by Targowski et al. that extensiveness of tobacco smoking correlated positively with increases in telomerase activity in tumor cells from patients with non small cell carcinoma of the lungs [100]. All these studies point to the fact that enhancement of the telomerase activity by cigarette smoke certainly underlies the cancer promotion potentials of cigarette smoke. However, which components in cigarette smoke altered telomerase activity are still not known. Further study in this aspect is very much needed.

### 2.5. Effects of Cigarette Smoke on Mobilization of Cellular Resources

Tumorigenesis requires adequate ability for protein synthesis and the energy for activating signaling. Indeed, there are indications that certain protein synthesis and mitochondria play central roles in neoplastic transformation. It is well known that mTOR and MAP kinase signaling pathways modulate the phosphorylation of transcriptional factors, stability of mRNAs, and protein synthesis [101]. Jin et al. reported that both nicotine and its metabolite NNK can induce survivin mRNA expression through AKT-mTOR and mediated *de novo* synthesis of survivin protein in normal lung epithelial cell HBE cells. This induced survivin expression has been claimed to play a role in the malignant transformation of HBE cells by stimulating the survival pathways [102]. 

Cigarette smoke may damage respiratory chain function in mitochondria enhancing oxidative stress leading to mitochondria dysfunction [103, 104]. It has also been reported that nicotine exposure resulted in reduced pancreatic mitochondrial enzyme activity, degranulation of beta cells, elevated islet oxidative stress, and impaired glucose stimulated insulin secretion in rats [105]. Continued exposure to ROS and free radicals from such “mitochondrial stress” may lead to mitochondria DNA (mtDNA) mutation which may play an important role in carcinogenesis [106]. Analyzing clinical samples, Tan et al. demonstrated mtDNA mutation in buccal cells of smokers [107]. Petros et al. also showed that tumor cells with mtDNA mutations grow faster then cells without mitochondrial mutation [108]. Hence, it is apparent that cigarette smoke would induce oxidative damage to the mtDNA leading to more aggressive tumor growths. Impact of cigarette smoke or its components on mitochondrial dysfunction needs further exploration.

**Step 3 (Cancer Progression)**

The “malignancy” of a tumor is usually evaluated by its ability in invasion and metastasis as well as in the associated angiogenesis. There are ample evidence which indicate that cigarette smoke participates in the processes of angiogenesis, invasion, and tumor metastasis. These phenomena are presented and discussed below.

## 3. Effects of Cigarette Smoke on Sustained Angiogenesis

Angiogenesis, the development of new blood vessels from endothelial cells (ECs), is a critical event which allows the cancer cells to receive adequate nutrients and oxygen. Angiogenesis involves mature vascular changes, including detachment of pericytes, degradation of extracellular matrix, endothelial cells remodeling, proliferation, migration, and formation of new endothelial cells into tubular structures [109]. Survival and proliferation of vascular endothelial cells are often stimulated by tumor-derived mitogens, and vice versa. Tumor cells are known to activate angiogenesis by changing the balance of angiogenic inducers such as VEGF (vascular endothelial growth factor) and bFGF (basic fibroblast growth factor), and by countervailing inhibitors such as thrombospondin-1 [29]. VEGF promotes angiogenesis and lymphangiogenesis in tumors, providing routes for dissemination. It has been shown that nicotine can induce angiogenesis both* in vitro* and* in vivo* and contributes to the growth of tumors [30, 110]. Similar to the FGF, nicotine is found to have the ability to promote migration, proliferation, tube formation and nitric oxide (NO) production of endothelial cells [111]. NO is a well-known vasodilator and angiogenesis mediator, and nicotine has been reported to enhance the expression of endothelial nitric oxide synthetase and promote NO release [110]. 

Nicotine is also found to induce expression of endothelial growth factors such as VEGF, bFGF, PDGF, TGF-*α*, and TGF-*β* in endothelial cells and smooth muscle cells [112, 113]. Enhanced bFGF release and increases in metalloproteinase expression with degradation of ECM have been demonstrated with nicotine [114, 115]. Moreover, nicotine is found to induce secretion of prostacyclin which is a vasodilating molecule associated with endothelial cell proliferation, survival and migration [116]. These effects are believed to be associated with cigarette smoke-induced hyperplasia of the intima in the blood vessels and other vascular wall lesions [115].

Tumor angiogenesis can also be modulated by the nAChR [117]. *α*7 nAChR is important in both physiological and pathological angiogenesis [110, 118]. *α*7 nAChR in endothelial cells needs to be sensitized or activated by hypoxia or ischemia in order to induce angiogenesis [110]. Indeed, specific antagonist of the *α*7 nAChR (*α*-bungaratoxin) is shown to inhibit nicotine-induced angiogenesis (new vascular tube formation from endothelial cells) [25, 114]. Interestingly enough, it is apparent that the AKT pathway is found to be not involved in either angiogenesis or VEGF release induced by nicotine [25]. In contrast, Heeschen et al. suggested that inhibition of ERK1/2, p38 MAPK, and PI3K/AKT can completely block and prevent endothelial tubule formation induced by nicotine-triggered *α*7 nAChR activation [110]. Consistent with Heeschen's study, Zhang and coworkers reported that nicotine apparently increases angiogenesis and invasion by activating PKC, PI3K/AKT, ERK1/2, mTOR, and Src in human NSCLC [119]. Excellent reviews on angiogenesis induced by nicotine were recently published [120, 121] and will not be further discussed here.

Interaction between nAChR and the growth factor-mediated angiogenesis occurs at signaling and transcription levels. Nicotine-induced expression of VEGF has been shown to be regulated by EGFR transactivation and via the ERK1/2 pathway in smooth muscle cells [122]. Phosphorylation of the VEGF receptor KDR by nicotine activates VEGF and increases its activity [112]. Additionally, nicotine can also upregulate the expression of VEGF receptor VEGFR2 during angiogenesis in certain cancer cells [123]. Recent study further indicated that nicotine can synergistically promote the proangiogenic effect of estradiol in nonsmall lung cancer [124]. Induction of angiogenesis in colon cancer by nicotine via *β*-AR followed by arachidonic acid pathway has also been reported [32, 125]. 

In sum, *α*7 nAChR subtype has been linked to angiogenic process induced by nicotine leading to tumor vascularity, inflammation, and ischemia. Nevertheless, whether nicotine or NNK acts specifically via nAChR or *β*-AR receptors or both or whether it is controlled in a cell-specific manner needs further study. Other components present in cigarette smoke that may also contribute to angiogenesis remain to be identified. The significant role of nAChR in various pathogenic angiogenesis is still largely unknown. This information would be critical for the development of new anti-angiogenic therapies. Several excellent reviews on the roles of nicotine and nAChR in angiogenesis exist [117, 120, 121, 126]. Readers are encouraged to refer to them for more detailed information.

## 4. Effects of Cigarette Smoke on Cancer Invasion and Metastasis

The ability of invasion and metastasis allows cancer cells to escape from the primary tumor mass to new terrains in the body. Metastasis is the final and most devastating consequence in malignancy. The processes of invasion and metastasis are exceeding complex. The genetic and biochemical determinants as well as the molecular mechanisms involved are still poorly understood. Many evidence indicate that cigarette smoking not only increases proliferation of cancer cells but also promotes metastasis [127]. Clinical and epidemiological studies suggest that smokers have more rapidly progressing tumors and cancer metastasis than non-smokers [128]. These processes are now known to be dependent on cellular and stromal interactions and on extracellular matrix degradation. E-cadherin is a cell-to-cell interaction molecule expressed on epithelial cells. A loss of E-cadherin is seen in epithelial to mesenchymal transition (EMT), which is a major pathologic event in cancer metastasis. Chronic treatment of nicotine downregulated the expression of ECM proteins such as E-cadherin and *β*-catenin with concomitant increases of fibronectin and vimentin in lung cancer cells [129]. Wei et al. also indicated that NNK enhanced colon cancer cell migration with downregulation of E-cadherin. This author also found that the expressions of Snail and ZEB1, 2 major transcription repressors of E-cadherin, were also induced by NNK in colon cancer cell cultures [44]. Contactin-1 is a glycophosphotidylinositol (GP)-anchored adhesion molecule. Its upregulation is significantly linked with tumor progression, metastasis and poor prognosis in lung cancer patients [130]. It has been shown that NNK can upregulate contactin-1 via *α*7 nAChR/ERK activation and enhances invasiveness of lung cancer cells [131].

The second general mediators for invasion and metastasis are the extracellular proteases [29]. Breakdown of the extracellular matrix (ECM) through a family of enzyme called matrix metalloproteinases (MMPs) is needed for tumor cells to invade adjacent tissue and to metastasize. Zong et al. reported that nicotine enhanced the invasiveness of esophageal squamous carcinoma cells (TE-13) by up-regulating the expressions and activity of MMP-2, and COX-2 [132]. Nicotine is found to enhance the activity of MMP-2, and MMP-9 as well as activation of plasminogen activators in a COX-2 and VEGF-dependent manner [123]. Osteopontin (OPN) is a proinflammatory and pro-metastatic protein. It can be upregulated by nicotine. It serves as a good marker for PDAC (pancreatic ductal adenocarcinoma) metastasis especially in cigarette smoking population [133]. In a recent investigation, Lazar et al. demonstrated that nicotine contributes to PDAC metastasis through the induction of MMP-9 and VEGF mediated by OPN [134]. 

PAHs, including BaP, are also found to play a role in the promotion of cancer metastasis. Through augmented COX-2 expression and PGE2 production via activated AhR pathway, BaP induces breast cancer cell invasions [135]. BaP and PAHs mixture has also been demonstrated to induce cancer cell invasions and metastasis through upregulating the expressions of MMPs, proteinase-activated receptor-2, fibronectin, migration stimulating factor, and Bcl-2 protein in lung adenocarcinoma [136]. The importance of FGF-9 and its up-regulation by BaP in lung cancer invasion and metastasis has been proposed. Indeed, recent study by Ueng et al. [137] demonstrated that BaP increases the invasive potential of lung cancer cells *in vitro*. Such process involves the up-regulation of FGF-9 mRNA expression via the p38 and ERK1/2 pathways [137]. 

During metastasis, the cancer cells co-opt signals that control leukocyte trafficking and chemokines-mediated cell migration [138]. Among these chemokines, CXCR4 and its natural ligand CXCL12 serve as key mediators for tumor migration and metastasis [139]. Nicotine has been shown to increase the expressions of several CXC chemokines receptors such as CXCR2, CXCR3, and CXCR4 as well as CCL12 in SCLC cells [140] suggesting the nicotine would stimulate cancer cell migration and eventual metastasis. 

Although epidemiology studies have long demonstrated the relationship between smoking and cancer metastasis, the molecular mechanisms of metastasis influenced by cigarette smoke or its components remain very limited. Further studies in this subject are urgently needed.

## 5. Summary, Conclusive Remarks, and Future Perspectives

In this paper, we have reviewed the recent investigations concerning cigarette smoke and cancer development, promotion and progression. While chemicals with carcinogenic potentials in cigarette smoke are many (over 62), most research efforts have been devoted to three components of cigarette smoke: nicotine, NNK, and PAHs. While PAHs are common chemicals in the environment, nicotine and NNK are considered to be tobacco specific. These three important components of cigarette smoke, especially nicotine and NNK, therefore, are targeted as the major compounds of focus in this review. Many previous reviews have devoted to the interrelationship between cigarette smoke and lung carcinogenesis or the genotoxicity of cigarette smoke or its components. In this review, we are focused on the mechanistic information on tumorigenesis, especially those involving epigenetic or non-gentoxic effects. Aside from lung cancer, other tobacco-related cancers are also discussed. It is our hope that this review will summarize the vast information cumulated in the literature and provide valuable reference resource for those who are interested in tobacco-related carcinogenesis. 

The overall mechanisms on carcinogenesis cancer promotion and progression are complex involving many molecular targets which include receptors, cell cycle regulators, mitogen-activated protein kinases, apoptosis mediators, angiogenic factors, and invasion, and metastasis mediators. Among the receptors, nAChR, *β*-AR, and AhR probably are the most important and have the closest association with cigarette smoke-induced carcinogenesis. Overexpression or activation of these receptors may result in the release of neurotransmitters and growth factors that participate in apoptosis inhibition, cell proliferation, angiogenesis, cancer cell invasion and metastasis. It should be noted that the importance of nAChR in cancer may be cell-type-dependent or specific and their sensitivity and expression can be also be modified by various environmental factors such as insecticide organophosphates [141]. 

**Figure 1:**
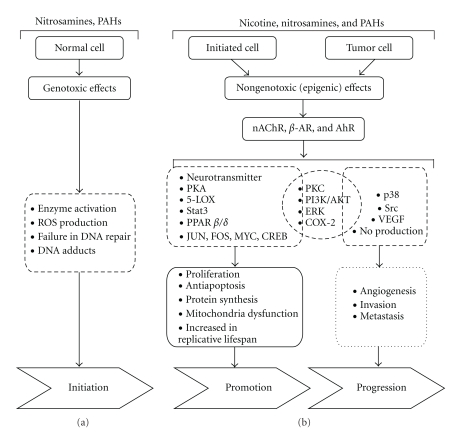
Diagrammatic models summarizing simplified molecular mechanisms of genotoxic and non-genotoxic modes of action in carcinogenesis by cigarette smoke. (a) Nitrosamines and PAHs are carcinogens, inducing genotoxic effects leading to cancer initiation. (b) Non-genotoxic (epigenic) effects of cigarette smoke components (nicotine, nitrosamines, and PAHs) in cancer promotion and progression. Activation of nAChR, *β*-AR, or AhR, followed by neurotransmitters release, activation of signaling pathways (PKA, 5-LOX, Stat3 and PPAR*β*/*δ*), and increased the expression of transcriptional factors (JUN, FOS, MYC, and CREB) regulate cancer promotion by cigarette smoke. PKC, PI3K/AKT, ERK, and COX-2 signaling pathways downstream of receptors play important roles in both promotion and progression stages. p38, Src, VEGF, and NO releasing involve in enhancement of cancer progression by cigarette smoke.

As shown in Figure 1, signaling pathways, PI3K/AKT, Stat3, and ERK1/2 play important roles in the carcinogenesis processes. They are also common paths affected by the cigarette smoke components, including nicotine, NNK, and PAHs. In addition, PKC, AKT, ERK, and COX-2 signaling pathways are involved in both promotion and progression stages by cigarette smoke. It is suggested that these molecules could be utilized the potential targets for future developments in cancer diagnoses or therapies.

Avoidance of cigarette smoke remains to be the best way of prevention for cigarette-related cancer. However, in view that tobacco smoke is legalized and smokers are still abundant, understanding on the health impacts by tobacco smoking still constitutes important public health concern. Understanding the disease process and the mechanisms involved is the first step to solution. The emerging understanding on the molecular mechanisms in the development and progression of caners induced by cigarette smoke provides novel inspirations and approaches for potential measures on early diagnosis, reduction in progression and metastasis, and therapy of cancers. Many dietary supplements, foods, or herbal medicines might significantly attenuate the proliferative effects by cigarette smoke. They may also enhance antigrowth signals to reduce cancer growth. From our own experience, the natural compound pterostilbene could induce apoptosis and autophagy in chemoresistant bladder cancer cells derived from nicotine exposure [142]. Future research on natural compounds may help to provide additional novel chemopreventive or chemotherapeutic possibilities in reducing cancer risks or other health impacts of cigarette smoke. This area of research is still weak and should be explored. 

This review has also discussed the various molecular mechanisms and paths involved in carcinogenesis induced by cigarette smoke. However, there are still many mysteries in the carcinogenic process by cigarette smoke. Several recommendations can be offered for future research needs.

1. In the past, most research efforts were focused on the proliferative and antiapoptosis mechanisms induced by cigarette smoking. As tumors are the results of multiple and interactive genetic abnormalities, study of cancers induced by cigarette smoke should assess more than one or two acquired alterations or paths. Explorations of other “paths” or mechanism other than those “popular” ones are needed.
2. Those molecular pathways which are significantly activated by cigarette smoke are probably the most important ones involved in cigarette smoke-induced tumorigenesis. These pathways include nAChR signaling (such as *α*7 nAChR, *α*9 nAChR, or *α*4/*β*2 nAChR), *β*-AR signaling, PI3K/AKT signaling, ERK1/2 signaling, Stat3 signaling, VEGF, and MMPs pathways, and so on. Targeting to modulate these pathways via dietary factors or therapeutic drugs may reduce cigarette smoking induced tumorigenesis significantly. Studies on the non-genotoxic (epigenetic) effects of cigarette smoke components are few and need more efforts. The epigenetic effects of cigarette component must be evaluated to include both upstream and downstream pathways.
3. Carcinogenesis is often species or cell-type specific and can be influenced by many factors or cofactors. Proper study of carcinogenicity requires consideration of these different variables. The same factor which is highly oncogenic to certain cell type or individuals may not be oncogenic to others. Moreover, some cell type may become susceptible to a “carcinogen” only in the presence of certain factor(s), co-factor(s), genetic predisposition, or immune depression. Identification of such influencing factors will be important. Specific “mechanism” for carcinogenesis for the same “carcinogen” may also vary in different tissues. Information obtained will be helpful for future cancer prevention, diagnosis and treatment.
4. Synergistic interaction between cigarette smoke components and other environmental toxicants or carcinogens, such as arsenic or dioxin, on cancer development has been demonstrated both epidemiologically and in animal studies [143–145]. Traditionally, most past investigations focused only on “single” compound or one cigarette smoke component. The synergistic interaction between otherwise “safe” level of environmental chemical and low level of cigarette smoke or its component (via either active or secondhand smoking) for carcinogenesis raised novel public health concerns and challenging questions. This area of research certainly deserves future attentions and efforts.

In conclusion, we have provided an overview on the major concepts and insights on the molecular mechanisms involved in cigarette smoke-induced cancers. It is hoped that these mechanistic insights can be translated into practical applications for the prevention and treatment of cigarette smoke-related cancers. We have also offered several recommendations for future research. We also hope that these suggestions will be helpful to those who are interested in this area of cancer research.
